# S100A4 drives non-small cell lung cancer invasion, associates with poor prognosis, and is effectively targeted by the FDA-approved anti-helminthic agent niclosamide

**DOI:** 10.18632/oncotarget.8969

**Published:** 2016-04-25

**Authors:** Rachel L. Stewart, Brittany L. Carpenter, Dava S. West, Teresa Knifley, Lili Liu, Chi Wang, Heidi L. Weiss, Tamas S. Gal, Eric B. Durbin, Susanne M. Arnold, Kathleen L. O'Connor, Min Chen

**Affiliations:** ^1^ Markey Cancer Center, University of Kentucky, Lexington, KY 40506, USA; ^2^ Department of Pathology and Laboratory Medicine, University of Kentucky, Lexington, KY 40506, USA; ^3^ Department of Molecular and Cellular Biochemistry, University of Kentucky, Lexington, KY 40506, USA; ^4^ Department of Toxicology, Guangdong Province Hospital for Occupational Disease Prevention and Treatment, Guangzhou, Guangdong, 510300, PR China; ^5^ Department of Biostatistics, University of Kentucky, Lexington, KY 40506, USA; ^6^ Division of Biomedical Informatics, University of Kentucky, Lexington, KY 40506, USA; ^7^ Department of Toxicology and Cancer Biology, University of Kentucky, Lexington, KY 40506, USA

**Keywords:** metastasin-1, NF-κB, MMP9, FSP-1, NSCLC

## Abstract

S100A4 (metastasin-1), a metastasis-associated protein and marker of the epithelial to mesenchymal transition, contributes to several hallmarks of cancer and has been implicated in the progression of several types of cancer. However, the impacts of S100A4 signaling in lung cancer progression and its potential use as a target for therapy in lung cancer have not been properly explored. Using established lung cancer cell lines, we demonstrate that S100A4 knockdown reduces cell proliferation, invasion and three-dimensional invasive growth, while overexpression of S100A4 increases invasive potential. In patient-derived tissues, S100A4 is preferentially elevated in lung adenocarcinoma. This elevation is associated with lymphovascular invasion and decreased overall survival. In addition, depletion of S100A4 by shRNA inhibits NF-κB activity and decreases TNFα-induced MMP9 expression. Furthermore, inhibition of the NF-κB/MMP9 axis decreases lung carcinoma invasive potential. Niclosamide, a reported inhibitor of S100A4, blocks expression and function of S100A4 with a reduction in proliferation, invasion and NF-κB-mediated MMP9 expression. Collectively, this study highlights the importance of the S100A4/NF-κB/MMP9 axis in lung cancer invasion and provides a rationale for targeting S100A4 to combat lung cancer.

## INTRODUCTION

A majority of lung cancer patients present at an advanced stage, which often precludes treatment with surgical resection alone [1]. Non-small cell lung cancer (NSCLC) accounts for roughly 85% of all lung cancers, and includes multiple histologic subtypes such as adenocarcinoma (ADC) and squamous cell carcinoma (SCC). Despite recent advances in screening and therapy, the prognosis for NSCLC remains poor, with only 15% of patients surviving five years after diagnosis [1]. A number of targeted agents show promise for the treatment of NSCLC, however, patients with advanced disease often develop resistance to these therapies [2, 3]. Therefore, there is an urgent need to better understand the crucial drivers of the metastatic process and to explore and develop novel therapeutic agents to reduce the morbidity and mortality associated with NSCLC.

S100A4 (also known as metastasin-1 (mts1)/fibroblast specific protein (FSP1)) is a calcium binding EF-hand protein that has been implicated in carcinoma progression and is a marker of the epithelial to mesenchymal transition (EMT) [4–6]. S100A4 has been identified in the nucleus, cytoplasm and extracellular space, suggesting that it signals through both intracellular and extracellular mechanisms [7]. Intracellular S100A4 interacts with a number of target proteins, such as p53, [8] and the heavy chain of non-muscle myosin IIA (MHC-IIA) [9]. We have shown that S100A4 interacts with Rhotekin to facilitate the formation of cell membrane protrusions and promote invasive growth in carcinoma cells [7]. S100A4 is secreted by both cancer and stromal cells to participate in both paracrine and autocrine signaling through its putative receptor RAGE, as well as through EGFR- and Toll-like receptor-4 (TLR-4)-mediated pathways. S100A4 can also activate NF-κB, thus stimulating a pathway that promotes proliferation and cell survival in multiple tumor types [10]. Collectively, through these intracellular and extracellular actions, S100A4 contributes to several hallmarks of cancer, including cell survival and proliferation, angiogenesis, invasion and metastasis, and tumor-promoting inflammation [11, 12]. Through these extensive biological functions, S100A4 expression is associated with tumor progression and is identified as a prognostic indicator in many human malignancies [6].

Despite extensive investigation of S100A4 in carcinoma progression, the impact of S100A4 signaling in lung cancer is poorly defined. In this study, we investigated the contribution of S100A4 in lung cancer cell invasion, determined the clinical significance of S100A4 expression in patient-derived lung cancer tissues, and provided a rational for targeting S100A4 signaling through repurposing an FDA-approved drug.

## RESULTS

### S100A4 drives the invasive potential of lung cancer cells

**Figure 1:**
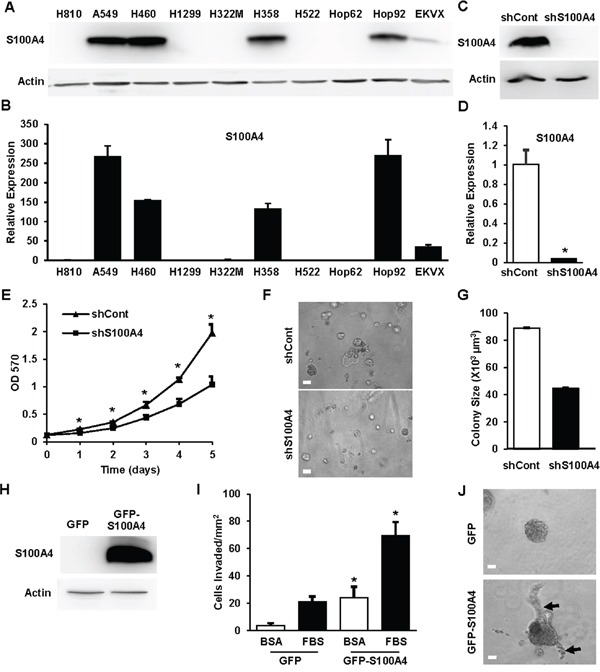
S100A4 drives the invasive potential of lung cancer cells **A** and **B.** Lung cancer cells as indicated were lysed in RIPA buffer, total cell lysates (80μg) were subjected to 15% SDS-PAGE, transferred and immunoblotted with rabbit anti-S100A4 antibody (A). RNA was isolated from cells and quantitative real time PCR (Q-PCR) was used to assess S100A4 expression levels (B). **C-G.** A549 cells with stable knockdown of S100A4 by shRNA targeting S100A4 (shS100A4) or expressing a non-targeting control (shCont) were generated. S100A4 expression was assessed by immunoblot analysis (C) and Q-PCR (D). Cell proliferation in standard (2D) culture was assessed by MTT (E). Cells were grown in 3D Matrigel for 5 days and representative phase contrast images for control and knockdown cells are shown (F). The diameter of 70-120 colonies from randomly chosen fields was measured, quantified for average colony volume and presented in (G). **H-J.** H1299 cells, stably transfected with pIRES-GFP-S100A4 (GFP-S100A4) or pIRES-GFP alone (GFP), were assessed for S100A4 expression by immunoblot analysis (H) or for invasion toward 1% FBS overnight (I) or grown in 3D Matrigel for 5 days (J). Representative data from at least three independent experiments are shown. Error bars represent the SEM of the mean in (G) and the SD of the mean from at least three replicates in (B, D, E and I). Arrows indicate invasive growth. Scale bar in (F and J) = 50μm. * indicates p<0.05.

We used a panel of established lung cancer cell lines with different genetic backgrounds (Supplementary Table 1) to examine S100A4 expression in lung cancer cells by immunoblot (Figure 1A) and Q-PCR (Figure 1B). S100A4 was highly expressed in about 50% of cell lines tested (Supplementary Table 1). Culturing cells in 3D Matrigel is commonly used to assess physiologically relevant tumorigenesis, morphogenesis and invasive potential [13, 14]. To test the contribution of S100A4 to lung cancer cell proliferation and invasive potential, loss-of function and gain-of function studies were performed in combination with 3D Matrigel analyses. S100A4 was depleted in A549 and H460 cells using lentiviral-based shRNA. Cells were grown in 3D Matrigel with growth media containing 2% FBS. Efficient knockdown of S100A4 in A549 cells (Figure 1C and 1D) dramatically decreased cell proliferation (Figure 1E) and invasive growth in the 3D Matrigel (Figure 1F and 1G). Similar results were observed in H460 cells (Supplementary Figure 1) and H358 cells (Supplementary Figure 2). In contrast, overexpression of S100A4 in H1299 cells promoted cell invasion and invasive growth in the 3D system (Figure 1H-1J) but did not increase cell proliferation (data not shown), suggesting that the effect of S100A4 in promoting proliferation is cell context-dependent. Collectively, these data demonstrate that S100A4 drives an invasive phenotype in lung cancer cells.

### S100A4 is overexpressed in patient-derived lung adenocarcinomas and associates with poor prognosis

**Figure 2:**
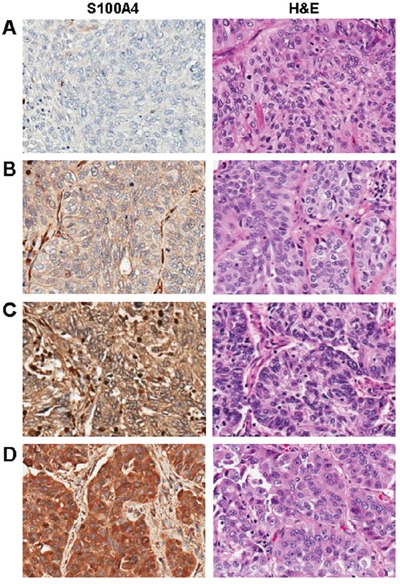
S100A4 expression patterns in non-small cell lung carcinoma **A-D.** Examples of negative (A), weak (B), moderate (C), and strong (D) S100A4 expression in non-small cell lung cancers (left panels), with corresponding hematoxylin and eosin stained (H&E) sections (right panels). Magnification = 200X for all images.

**Figure 3:**
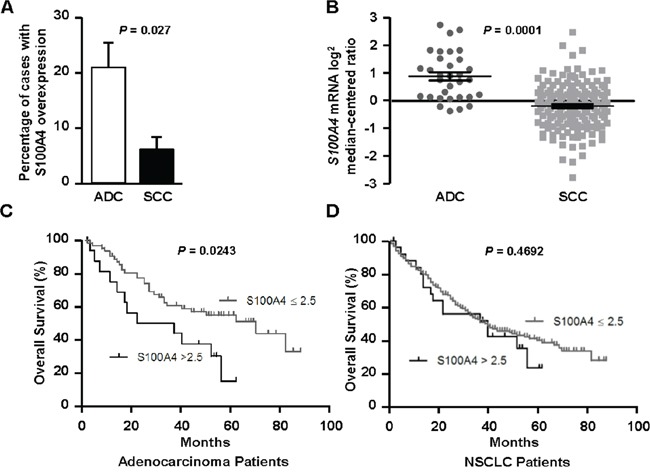
S100A4 is overexpressed in lung adenocarcinoma, where it associates with decreased overall survival **A-D**. Using semi-quantitative IHC data from our TMA, we found that S100A4 levels were significantly elevated in adenocarcinoma (ADC) when compared to squamous cell carcinoma (SCC, A), which was confirmed using an external gene expression dataset (B). In patients with lung adenocarcinoma, elevated expression of S100A4 (>2.5) correlated with shorter median survival (C). A significant relationship between elevated S100A4 expression and overall survival was not identified when examining all histologic subtypes (NSCLC) combined (D).

We constructed a lung cancer tissue microarray (TMA) and stained sections for S100A4 using immunohistochemistry in order to examine S100A4 expression in patient-derived tissues (*N* = 212). A wide range of staining intensities was observed in lung carcinoma cells, which were scored using a semi-quantitative scale ranging from 0 to 3 (Figure 2). In addition, positive staining was observed in lymphocytes and macrophages, which served as positive internal controls (data not shown). Next, we correlated S100A4 expression with clinical and pathological features. We found that S100A4 was preferentially overexpressed in lung adenocarcinoma when compared to squamous cell carcinoma (Figure 3A), which was confirmed using a publicly available gene expression dataset (Figure 3B). S100A4 overexpression was much less common among the other histologic subtypes as only 9.7% of these samples exhibited elevated levels of S100A4 expression (Supplementary Table 2). Furthermore, we found that S100A4 overexpression was associated with the presence of lymphovascular invasion (Table 1) and decreased overall survival among patients with lung adenocarcinoma (Figure 3C; median survival: 29.5 versus 70 months, hazard ratio 2.62, 95% confidence interval 1.133 to 6.035, *P* = 0.0243). When all histologic subtypes were combined, there was no significant difference in median survival between patients with and without S100A4 overexpression (Figure 3D; hazard ratio 1.220, 95% confidence interval 0.6904 to 2.157, *P* = 0.4692). This observation suggests that S100A4 overexpression has a higher impact in the lung adenocarcinoma subpopulation when compared to the squamous cell carcinoma subpopulation.

**Table 1:** Clinico-pathologic parameters and S100A4 expression in lung adenocarcinoma patients

	S100A4 High (>2.5)	S100A4 Low (≤2.5)	*P* value
Adenocarcinoma	17/81 (21%)	64/81 (79%)	
Age at diagnosis			
≥ 65 years	10/38 (26%)	28/38 (74%)	*P* = 0.2893
< 65 years	7/43 (16%)	36/43 (84%)	
Collaborative Stage			
Stage I-II	10/53 (19%)	43/53 (81%)	*P* = 0.5556
Stage III-IV	6/24 (25%)	18/24 (75%)	
Lymph node metastasis			
N0	10/50 (20%)	40/50 (80%)	*P* = 0.7731
N1 or N2	6/26 (23%)	20/26 (77%)	
Lymphovascular invasion			
Present	10/25 (40%)	15/25 (60%)	*P* = 0.0080
Absent	7/56 (12.5%)	49/56 (87.5%)	
Pleural Invasion			
Present	6/33 (18%)	27/33 (82%)	*P* = 0.7825
Absent	11/48 (23%)	37/48 (77%)	

### Niclosamide, an FDA-approved drug, targets S100A4 to abbrogate the invasive potential of lung cancer cells

**Figure 4:**
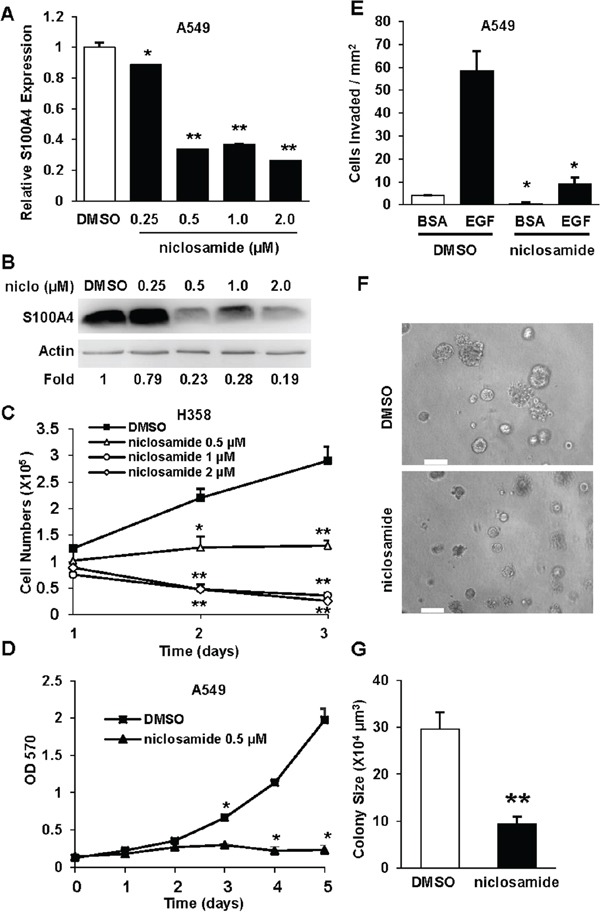
Niclosamide decreases S100A4 expression, lung cancer cell proliferation, invasion and invasive growth **A** and **B.** A549 cells were treated with niclosamide at the indicated concentrations for 72 hrs, and S100A4 expression was assessed by Q-PCR (A) or immunoblotting analysis (B). **C** and **D.** Cells were treated with varied concentrations of niclosamide for 1-5 days and then cell number assessed by either direct cell count (C, H358) or MTT assay at various time intervals (D, A549). **E.** A549 cells were treated with niclosamide at the indicated concentrations for 3 days, trypsinized and assessed for EGF-stimulated (5 ng/ml) Matrigel invasion in the presence or absence of niclosamide. **F, G.** A549 cells were cultured in 3D Matrigel with or without 1 μM niclosamide for 6 days. Representative phase contrast images are shown (F). The diameter of 70-120 colonies from randomly chosen fields was measured, quantified for average individual colony volume (G). Representative data from at least three independent experiments are shown. Error bars represent the SEM of the mean in (G) and the SD of the mean from at least three replicates in (A, C, D and E). Scale bar in (F) = 100 μm. * indicates p<0.005. ** indicates p<0.0001.

Niclosamide affects multiple signaling pathways that are important in cancer progression and has also been shown to block S100A4 expression in colon cancer cells [15, 16]. Our data show that S100A4 drives an invasive phenotype in lung cancer cells (Figure 1), thus positioning S100A4 as a potential target for the treatment of advanced NSCLC. Therefore, we investigated whether niclosamide also suppresses S100A4 expression in lung cancer cells and whether it inhibits S100A4-mediated functions. Niclosamide blocked S100A4 expression in lung carcinomas both at the mRNA (Figure 4A) and protein levels (Figure 4B) in a dose-dependent manner. This inhibitory effect of niclosamide on lung cancer cell proliferation was also investigated using H358 and A549 cells treated with varied concentrations of niclosamide. We performed direct cell count or MTT assay to assess the number of viable cells over 3-5 days. Niclosamide treatment dramatically decreased the proliferation of H358 cells (Figure 4C) and A549 cells (Figure 4D), at concentrations as low as 0.5 μM. We then evaluated the effect of niclosamide treatment on the invasive capacity of A549 cells using Transwell invasion assays and by monitoring invasive growth in the 3D Matrigel system. As visualized in Figure 4E, niclosamide treatment decreased EGF-stimulated A549 cell invasion. Consistent with these observed effects on proliferation and invasion, niclosamide significantly inhibited invasive growth in the 3D Matrigel (Figure 4F and 4G). These data demonstrate that niclosamide blocks the invasive capabilities of lung cancer cells driven by S100A4. To analyze the specific contribution of S100A4 to anti-tumor activity of niclosamide, we performed an invasion assay as we did in Figure 1I on H1299 cells stably expressing a bicistronic S100A4-pIRES-EGFP vector in the presence and absence of nicosamide. In this assay S100A4 expression level is not subject to regulation by niclosamide. We found that overexpressing S100A4 did not prevent the inhibitory effect of niclosamide, instead, niclosamide significantly inhibited S100A4-mediated invasion to the basal level (Supplementary Figure 4). These results suggest that niclosamide inhibited lung cancer cell invasive potential is not only limited to suppression of S100A4 expression but also extended to inhibition of S100A4 signaling-mediated function.

### Inhibition of S100A4 decreases NF-κB activity

**Figure 5:**
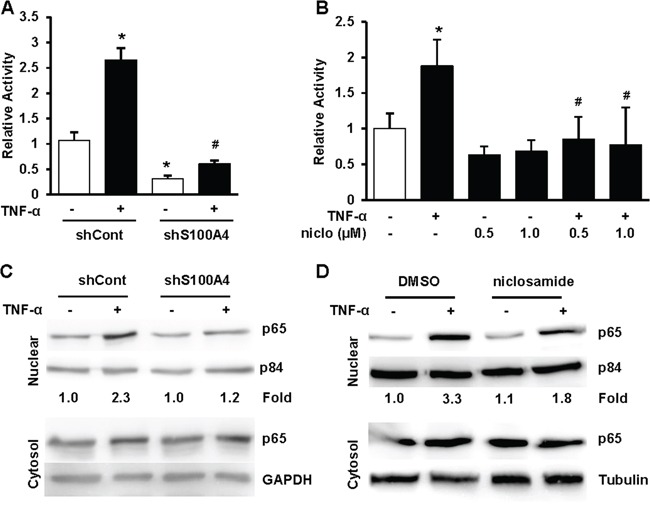
Inhibition of S100A4 decreases NF-κB activity **A** and **B.** A549 shCont and shS100A4 cells (A) or A549 parental cells (B) were transfected with NF-κB firefly luciferase reporter and TK-renilla luciferase control, treated with 5 ng/ml TNF-α with or without niclosamide at indicated concentrations for 24 hrs before harvesting for Dual luciferase activity assays. **C.** A549 shCont and shS100A4 cells (C) were serum starved overnight, then stimulated with 5 ng/ml TNF-α for 4 hrs before harvesting for cell fractionation. **D.** A549 parental cells were treated with 0.5 μM niclosamide for 48 hrs, serum starved in the presence or absence of niclosamide overnight, then stimulated with 5 ng/ml TNF-α for 4 hrs before harvesting for cell fractionation. Nucleic and cytosolic proteins were separated by SDS-PAGE, transferred and probed for p65, p84 and GAPDH. Representative data from at least three independent experiments are shown. Error bars represent the SD of the mean from three replicates. (*) and (#) in (A) indicate p<0.05 compared to control and TNF-α treated shCont cells, respectively. (*) and (#) in (B) indicate p<0.001 compared to control and TNF-α treated cells, respectively.

Considerable evidence demonstrates that NF-κB signaling is constitutively activated in solid tumors and is essential for lung cancer tumorigenesis, invasion and metastasis [17]. S100A4 has been shown to affect canonical NF-κB signaling through cross-talk with RAGE, TLR-4 and EGFR. In the canonical NF-κB pathway, p65 (RelA) and p50 form a heterodimer that is sequestered by IkB-α under unstimulated conditions. Stimulation with cytokines, such as TNF-α, activates the IkB kinase (IKK) complex, which phosphorylates IkB-α, leading to its degradation. NF-κB is then released, phosphorylated and translocated into the nucleus where it activates a variety of genes that promote cell proliferation, survival and invasion [18, 19]. To test whether depletion of S100A4 inhibits the NF-κB pathway, we evaluated NF-κB activity using a luciferase activity assay and p65 nuclear translocation. Control (shCont) and S100A4 knockdown (shS100A4) A549 cells were transfected with an NF-κB luciferase reporter gene construct and treated with 5 ng/ml TNF-α. As shown in Figure 5A, knockdown of S100A4 not only decreased TNF-α induced NF-κB activity, but also reduced the basal activity of NF-κB. We then investigated whether depletion of S100A4 inhibits p65 nuclear translocation. As shown in Figure 5C, knockdown of S100A4 in A549 cells decreased p65 nuclear localization in response to TNF-α treatment. To determine the biological consequences of NF-κB inhibition, a Matrigel invasion assay was performed with TNF-α as the attractant in A549 shCont and shS100A4 cells. We observed that cells with S100A4 knockdown (shS100A4) displayed decreased invasive potential (Supplementary Figure 3). Since niclosamide treatment suppressed S100A4 expression and decreased the invasive potential of lung cancer cells (Figure 4), we hypothesized that treatment with niclosamide would block NF-κB activity. To test this hypothesis, we performed an NF-κB luciferase activity assay and p65 nuclear translocation in response to TNF-α with and without niclosamide treatment. Niclosamide treatment decreased NF-κB luciferase activity and inhibited p65 nuclear translocation in A549 cells (Figure 5B and 5D). Similar results were seen using H460 cells (data not shown). Taken together, these data show that S100A4 drives lung cancer invasion by activating NF-κB activity. These data also suggest that the inhibitory effect of niclosamide on NF-κB activity could be, at least in part, due to S100A4 suppression.

### S100A4 is required for TNF-α-induced and NF- κB-mediated MMP9 expression

**Figure 6:**
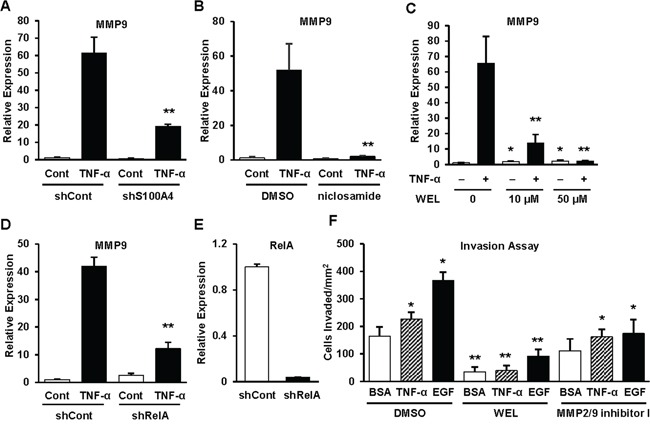
The S100A4/NF-κB/MMP9 axis is critical to the invasive capacity of lung cancer cells **A-C.** Cells were exposed to a variety of experimental conditions and then TNF- α was added for an additional 24 hrs prior to assessment of MMP9 expression by Q-PCR. A549 shCont and shS100A4 cells treated with 5 ng/ml TNF-α for 24 hrs (A), or parental A549 cells were pretreated with 1μM niclosamide for 48 hrs (B), or parental A549 cells treated with 10 μM and 50 μM Wedelolactone (WEL) for 24 hrs (C). **D-E.** A549 shCont and shRelA cells were treated with 5 ng/ml TNF-α for 24 hrs, then harvested and assessed for MMP9 (D) and RelA (E) expression by Q-PCR. **F.** Parental A549 cells were pretreated with 10 μM Wedelolactone or 100 μM MMP2/9 inhibitor I for 24 hrs, trypsinized, rinsed and assessed for Matrigel invasion toward 5 ng/ml TNF-α or 5 ng/ml EGF overnight in the presence or absence of the respective inhibitors. Representative data from at least three independent experiments are shown. Error bars represent the SD of the mean from three replicates.* indicates p<0.05, ** indicates p<0.01.

TNF-α induces MMP9 expression through the NF-κB pathway [20]. We tested whether inhibition of S100A4 affects downstream targets of NF-κB such as MMP9. We used Q-PCR to assess the expression of MMP9 in A549 shCont and shS100A4 cells with and without TNF-α. Knockdown of S100A4 significally decreased basal expression of MMP9 in H358 (Supplementary Figure 2C) and A549 cells as well as TNF-α-induced MMP9 expression (Figure 6A). In addition, niclosamide treatment, which decreases S100A4 expression (Figure 4) and NF-κB activity (Figure 5), results in a significant inhibiton of TNF-α-induced MMP9 expression in A549 cells (Figure 6B). Collectively, these data support the assertion that S100A4 confers an invasive phenotype, at least in part, through the NF-κB pathway. To substantiate the finding that MMP9 expression depends on NF-κB, we blocked the NF-κB pathway by using the NF-κB inhibitor, wedelolactone, which directly inhibits the phosphorylation and degradation of IκBα, and by reducing RelA (p65) expression using a lentiviral-based shRNA. As shown in Figure 6C, pretreatment of A549 cells with wedelolactone prior to stimulation with TNF-α dramatically decreased MMP9 expression. Efficient RelA knockdown significantly abrogated TNF-α-induced MMP9 expression (Figure 6D and 6E).

### The NF-ĸB/MMP9 pathway is critical to the invasive potential of NSCLC cells

Our data demonstrate that S100A4 drives the invasive capacity of lung cancer cells, affects the NF-κB pathway, and acts as an essential component of TNF-α-induced and NF-κB-dependent MMP9 expression. Next, we investigated whether the NF-κB/MMP9 axis is critical to the invasive capacity of lung cancer cells. Accordingly, we pretreated A549 cells with inhibitors of either NF-κB or MMP2/MMP9, then performed Matrigel invasion assays. Inhibition of the NF-κB/MMP9 pathway not only decreased the basal invasive capacity but also inhibited cell invasion toward chemo-attractants such as TNF-α and EGF (Figure 6F). Collectively, these results implicate S100A4 in driving lung cancer invasive potential, at least in part, through activation of the NF-κB/MMP9 axis.

## DISCUSSION

The majority of patients with advanced NSCLC die within 18 months of diagnosis, usually as a result of metastatic disease [21, 22]. Elucidation of the mechanisms driving lung cancer invasion and metastasis can lead to the development of therapies targeting these pathways which, in turn, will have significant impacts on patient survival and quality of life. S100A4 is known to promote aggressive behavior in human cancers [6] and can be induced by cigarette smoke [Monzon ME, et.al., Am J Resp Crit Care. 2011; 183. Meeting Abstract]. S100A4 is often overexpressed in NSCLC as well as other tobacco-related malignancies, making it an excellent therapeutic target for lung and other cancers. As published in other reports [23, 24], we found that S100A4 expression was elevated in lung adenocarcinoma. However, due to the small sample size and lack of non-smokers in our population, we could not confirm an association between S100A4 expression and tobacco exposure. We found that in lung adenocarcinoma patients, S100A4 elevation was associated with reduced overall survival as well as lymphovascular invasion, a marker of poor prognosis in many cancers. A similar association between elevated S100A4 expression and lymphovascular invasion has previously been demonstrated in colorectal carcinoma [25]. Taken together, these data suggest that S100A4 could serve as a marker of poor prognosis as well as a valuble therapeutic target in lung cancer.

It is well established that S100A4 overexpression contributes to several hallmarks of cancer. Supported by a previous study [26], we found that depletion of S100A4 inhibited cell proliferation in both H358 and A549 cells. However, forced expression of S100A4 alone in H1299 cells is not sufficient to promote cell proliferation, suggesting that the role of S100A4 in cell proliferation could be cell context dependent. Recent studies demonstrate that S100A4 promotes lung cancer cell mortility and cell growth [27], although the detailed mechanisms have not been fully defined. Notably, extracellular S100A4 has been shown to activate canonical NF-κB pathway through activation of IκB-α [10, 28–31]. NF-κB signaling is constitutively activated in solid tumors including lung cancer [32] and is essential for lung cancer tumorigenesis, invasion and metastasis [17]. Although NF-κB has been considered as a therapeutic target, its role in normal physiology poses a substantial challenge for directly targeting NF-κB for cancer therapy. Therefore, alternatively targeting the upstream or downstream pathways that lead to constitutive NF-κB activation may be more sucessful [33]. Our data demonstrated that inhibition of S100A4 decreased NF-κB p65 nuclear translocation in response to TNF-α. We also found that depletion of S100A4 decreased the basal activity of NF-κB signaling. Inhibition of the NF-κB pathway dramatically blocked the invasive capacity of lung cancer cells, suggesting that inhibition of S100A4 signaling could effectively mitigate NF-κB-mediated effects.

Matrix metalloproteinases (MMPs) are essential in tumor invasion and metastasis due to their abilities to degrade the extracellular matrix (ECM) and also to change cellular behavior to modulate the tumor microenvironment. Downstream of the NF-κB pathway, we found that MMP9, but not MMP2, was dramatically inhibited by S100A4 depletion in response to TNF-α (data not shown). Importantly, MMP9 inhibition decreased the TNF-α- and EGF-stimulated invasiveness of A549 cells, highlighting the importance of the S100A4/MMP9 axis in S100A4-driven invasion in lung cancer cells. Given that S100A4 is an established metastasis-promoting protein, it is not surprising that this S100A4/MMP9 pathway is demonstrated in other cancer types [34]. In support of our study, recent work has shown that both S100A4 and MMP9 are upregulated in patient-derived lung cancer tissues and positively correlate with each other [23], further implicating the importance of S100A4/MMP9 signaling in lung cancer progression.

Currently, there is no clinically available treatment targeting S100A4 and its pleiotropic roles in cancer progression. Multiple strategies have been proposed to block S100A4 function in cancer. These strategies include targeting S100A4 with specific antibodies or interfering with the interaction of S100A4 and its targets using small molecular inhibitors [35, 36]. However, S100A4 is often present at high concentrations in malignancies, and is also high in inflammatory disorders [37, 38], which complicates the use of antibodies to inhibit S100A4 signaling extracellularly and/or through blocking the intracellular action. Niclosamide, an anti-helminthic agent used for over 50 years to treat tapeworm infections in humans, is proposed to have a favorable safety profile due to poor systemic absorption from the gastrointestinal tract [15]. However, animal studies have shown that the concentration of niclosamide in tumor tissue and plasma can reach up to 1 μM, thus revealing that it is readily absorbed without significant toxicity to normal fibroblasts and peripheral blood mononuclear cells [15, 39]. Niclosamide inhibits S100A4 through the Wnt/β-catenin pathway in colon cancer [16]. Our study extends current understanding by showing that at physiologically achievable levels, niclosamide can effectively target the S100A4/NF-κB/MMP9 signaling axis in lung cancer by decreasing S100A4 expression. This decrease in S100A4 expression in turn decreases NF-κB activity and NF-κB-mediated MMP9 expression. Given that S100A4 is secreted and can be detected in patient serum, our study suggests that S100A4 could be used as a potential biomarker to monitor the response to niclosamide treatment, although *in vivo* studies are needed to confirm this concept.

Our study highlights the important role of the S100A4/NF-κB/MMP9 signaling axis in promoting lung cancer invasive capacity, and demonstrates that S100A4 overexpression associates with reduced overall survival among patients with lung adenocarcinoma. Importantly, our study demonstrates that niclosamide dramatically inhibits the NF-κB/MMP9 signaling axis by suppressing S100A4 to block the invasive capacity of lung cancer cells. Since S100A4 promotes metastasis, our study provides strong evidence and rationale for targeting S100A4 by repurposing niclosamide to block invasion and prevent metastasis in NSCLC.

## MATERIALS AND METHODS

### Cell lines and reagents

Lung cancer cell lines that are representative of different subtypes of lung cancer were obtained from ATCC. Lung adenocarcinoma (A549, EKVX, H358, Hop62, H322M, H522, H838, and H23), large cell lung carcinoma (H460, Hop92), NCSLC (H1299, H810) and small cell lung cancer (H82) cell lines were used in this study. A549, H1299, H358 and H460 cells used for biological functional analysis were authenticated with short tandem repeat (STR) profile analysis by Genetica DNA laboratories in May 2015. Niclosamide and Wedelolactone were obtained from Sigma-Aldrich (St. Louis, MO). MMP2/9 inhibitor I was from Calbiochem/EDM Millipore (Billerica, MA). The pIRES-GFP-S100A4 construct was obtained from Dr. Masashi Fukayama (University of Tokyo, Tokyo, Japan) [40].

### Immunoblotting

Cells were harvested and lysed in RIPA buffer (150 mM NaCl, 0.5 mM EGTA, 0.5% sodium deoxycholate, 0.1% SDS, 1% Triton X-100, 50 mM Tris-HCl pH 7.4, 15 μg/ml protease inhibitor cocktail, 1 mM PMSF, 50 mM NaF and 10 mM sodium pyrophosphate). Total cell lysates (80 μg) were subjected to 10% or 15% SDS-PAGE, transferred and immunoblotted with rabbit anti-S100A4 antibody (Dako, Carpinteria, CA), p65 (Santa Cruz, Dallas, TX) or p84 (GeneTex, Irvine, CA). Tubulin and β-actin antibodies were from Sigma-Aldrich and were used as the loading controls.

### Three-dimensional (3D) culture

Culturing lung cancer cells in 3D was performed as described previously [14]. Briefly, cells (1 × 10^4^) in 200 μl growth media with 2% FBS were seeded onto solidified growth factor-reduced Matrigel (BD Biosciences, San Jose, CA; 100 μl per well of 8-well chamber slide) and then covered with medium containing 10% Matrigel. The next day, DMSO or niclosamide at the indicated concentration was added to the cultures. When the control cells developed an invasive growth phenotype (approx. 5-6 days), phase contrast images of randomly chosen fields were taken with a Nikon Ti-E inverted microscope and analyzed using Nikon Elements software.

### Matrigel invasion assay

Matrigel (10 μg, BD Biosciences) was dried onto the upper well of transwell chambers (6.5-mm diameter, 8-μm pore size, Corning, Corning, NY). One hour before the assay, Matrigel was reconstituted with 100 μl of serum-free medium and the bottom chamber was coated with 5 μg/ml fibronectin. Cells (70% confluent) were trypsinized and rinsed three times with serum-free medium plus 250 μg/ml BSA. Cells (5×10^4^) were added to the upper chamber, and medium/BSA containing 5 ng/ml EGF, 5 ng/ml TNF-α or 1% FBS (control) was added to the bottom chamber as indicated; cells were allowed to invade overnight at 37°C. Non-invaded cells were removed from the top chamber using a cotton swab; invaded cells on the bottom of the transwell membrane were fixed with 100% methanol and stained with 1% crystal violet. Four fields per well were counted and averaged, and the data were presented as the mean number of cells invaded per mm^2^ +/− standard deviation from triplicate determinations.

### Cell proliferation assays

Cell proliferation was assessed by direct cell counting or by 3-(4, 5-dimethylthiazol-2-yl)-2.5-diphenyltetrazolium bromide (MTT) assay. Cells (0.5 x10^5^) were seeded in each well of a 12-well plate for direct cell counting or cells (2×10^3^) were seeded in each well of a 96-well plate for MTT assay. For niclosamide treatment, fresh growth media containing niclosamide or DMSO was added starting the day after plating, and added daily thereafter. Cells were trypsinized and directly counted by using a Vi-Cell XR cell viability counter (Beckman Coulter, Brea, CA) at the indicated time points. Alternatively, MTT assay was performed as previously described [41].

### Quantitative real time PCR (Q-PCR)

Total RNA was extracted using TRizol reagent (Life Technologies/ThermoFisher, Grand Island, NY). Total RNA (1μg) was used to reverse-transcribe into cDNA using the High Capacity cDNA Reverse Transcription Kit (Applied Biosystems/ThermoFisher, Grand Island, NY). Expression of target genes was assessed by Comparative Ct (ΔΔCt) using commercially available probes and master mix reagent and performed on a StepOnePlusTM 96-well instrument as described by the manufacturer (Applied Biosystems). The expression level of each gene was normalized by 18S or β-actin RNA and reported as a relative level to a specified control, as noted.

### Stable lentiviral shRNA cell line generation

For stable reduction of S100A4 expression in A549 and H460 cells, lentivirus-mediated shRNA construct pLKO.1-puro targeting human S100A4 (S100A4-#A6) or containing non-targeting sequence (Sigma-Aldrich) were packaged into virus. In brief, the control or S100A4-specific shRNA construct was co-transfected with Mission lentiviral packaging mix (Sigma-Aldrich) into 293T cells using polyethylenimine (PEI) based on 1:3 ratio of DNA: PEI. The viral supernatant was collected 48 hrs after transfection, as described previously [7]. Then, A549 and H460 cells were infected with virus containing media and stable transfectants were selected with puromycin (2 μg/ml). The targeting sequences for human S100A4 are 5′ aagctcaaca agtcagaact aaa (#A6) and 5′CGCCATGATGTGTAACGAATT 3′ (#A8).

### Generation of S100A4 overexpression cell lines

H1299 cells in 10-cm dishes were transfected with 8 μg GFP control or pIRES-GFP-S100A4 constructs using Lipofectamine 2000 (Invitrogen/ThermoFisher, Grand Island, NY). Transfected cells were selected with G418 (400 μg/ml) and sorted for GFP by fluorescence activated cell sorting (FACS).

### Tissue microarray (TMA) construction and immunohistochemistry (IHC) staining

Institutional Review Board Approval (13-0692-P6H) was obtained prior to initiation of the project. Surgically resected NSCLC cases from 2006-2010 from our institution were screened for inclusion in this study. Cases were excluded if the primary tumor was not of lung origin or if inadequate pathological material existed. A total of 216 lung cancer cases were used to build the tissue microarrays (TMAs). A variety of histologic types were selected, including 83 adenocarcinomas and 102 squamous cell carcinomas (for complete list see Supplementary Table 2). Pathologic features (tumor grade, histologic type, presence of lymphovascular invasion etc.) were abstracted from pathology records and patient charts. Treatment and outcome data were collected by the Markey Cancer Center (MCC) Cancer Research Informatics Shared Resource Facility and the Kentucky Cancer Registry. Our pathologist then selected appropriate blocks and identified tumor areas on H&E stained slides for inclusion in the TMA. For each case, three 2-mm tissue cores were removed from each formalin-fixed, paraffin embedded (FFPE) archival tumor block, and were transferred to recipient paraffin blocks using a TMArrayer ^TM^ (Pathology Devices, Westminster, MD). Listing of randomly-sorted samples for allocation into the recipient TMA was generated by the MCC Biostatistics and Bioinformatics Shared Resource Facility, and then assembled into TMA blocks (12 in total) by the MCC Biospecimen and Tissue Procurement Shared Resource Facility. After construction, TMA blocks were heated at 45°C for 1 hr and then cooled to complete assembly. S100A4 expression was assessed by IHC staining as previously reported [7, 42] and scored by a pathologist blinded to clinical variables. Out of 216 cases included in the TMA, 4 were excluded from analysis due to folded or absent tissue cores. In the remaining 212 cases, a semi-quantitative scale was used to score S100A4 expression as follows: negative (0), weakly positive (1), moderately positive (2), and strongly positive (3) (Figure 2). Results from each of the three tissue cores were averaged together to produce a final score for each tumor. S100A4 overexpression was defined as a final score >2.5.

### NF-κB activity assays

For NF-κB luciferase reporter assays, cells grown in a 24-well plate were co-transfected with 0.25 μg of the NF-κB reporter (A gift from Dr. Yanan Tian, Texas A& M University) along with pRL-TK Renilla control reporter at a 50:1 ratio for 24 hrs. Then, cells were induced by 5 ng/ml TNF-α, vehicle control with or without niclosamide at the indicated concentration for 24 hrs. Then, cells were collected and luciferase activity was measured using the Dual-Luciferase Reporter Assay System (Promega, Madison, WI). These data are presented as a relative value to internal control or fold induction as individual control.

To detect NF-κB p65 nuclear translocation, cells were serum-starved overnight and then stimulated with 5 ng/ml of TNF-α for 4 hrs. For niclosamide treatment, cells were pretreated with DMSO (control) or 0.5 μM or 1 μM niclosamide for 48 hrs before serum starvation, then stimulated with 5 ng/ml of TNF-α for 4 hrs. Cell fractionation was then performed as described previously [41].

### Statistical analysis and data mining

For quantitative variables, differences between groups were analyzed using Welch's t-test or the Mann–Whitney U test, where appropriate. For categorical variables, differences between groups were analyzed using chi-squared or Fisher's exact test, where appropriate. All tests were two-tailed. Survival differences were assessed via log-rank test. Significance was reached when P < 0.05. Statistical analyses were performed using GraphPad software (La Jolla, CA). For data mining, a lung cancer gene expression dataset [43] generated by The Cancer Genome Atlas (TCGA Research Network, http://cancergenome.nih.gov/) was accessed and visualized using The Oncomine™ Platform (Life Technologies, Ann Arbor, MI).

## SUPPLEMENTARY FIGURES AND TABLES


