# Supplementary material for: S100A4 drives non-small cell lung cancer invasion, associates with poor prognosis, and is effectively targeted by the FDA-approved anti-helminthic agent niclosamide
Source: Oncotarget. 2016 Apr 25;7(23):34630–42. doi: 10.18632/oncotarget.8969 (PMC5085181; doi:10.18632/oncotarget.8969)
Supplement: Supplementary file 1 [file oncotarget-07-34630-s001.pdf]

SUPPLEMENTARY FIGURES AND TABLES

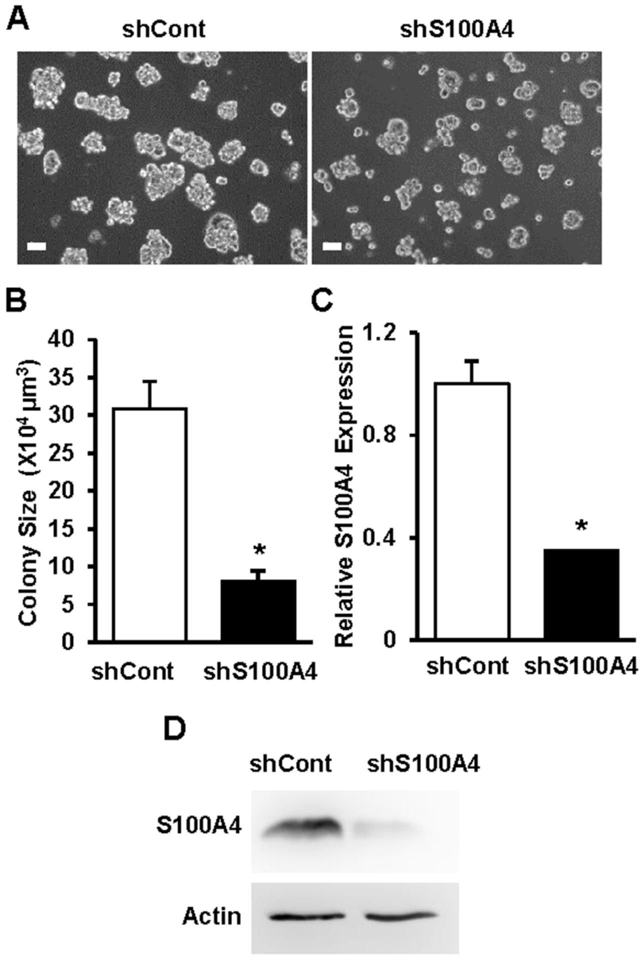

Supplementary Figure S1: Inhibition of S100A4 decreases the invasive potential in H460 cells. H460 cells with stable knockdown of S100A4 by shRNA targeting S100A4 (shS100A4) or expressing a non-targeting control (shCont) were grown in Matrigel (3D) for 5 days **A.** Representative phase contrast images. **B.** The diameter of 70-120 colonies from randomly chosen fields was measured, quantified for average colony volume. Error bars in (B) represent the SEM of the mean. Scale bar in (A) = 50µm. **C and D.** S100A4 expression was assessed by Q-PCR (C) and immunoblot analysis (D). Error Bars in (C) represent the SD of the mean. Data are representative from ≥ 3 independent experiments. * indicates $p<0.05$.

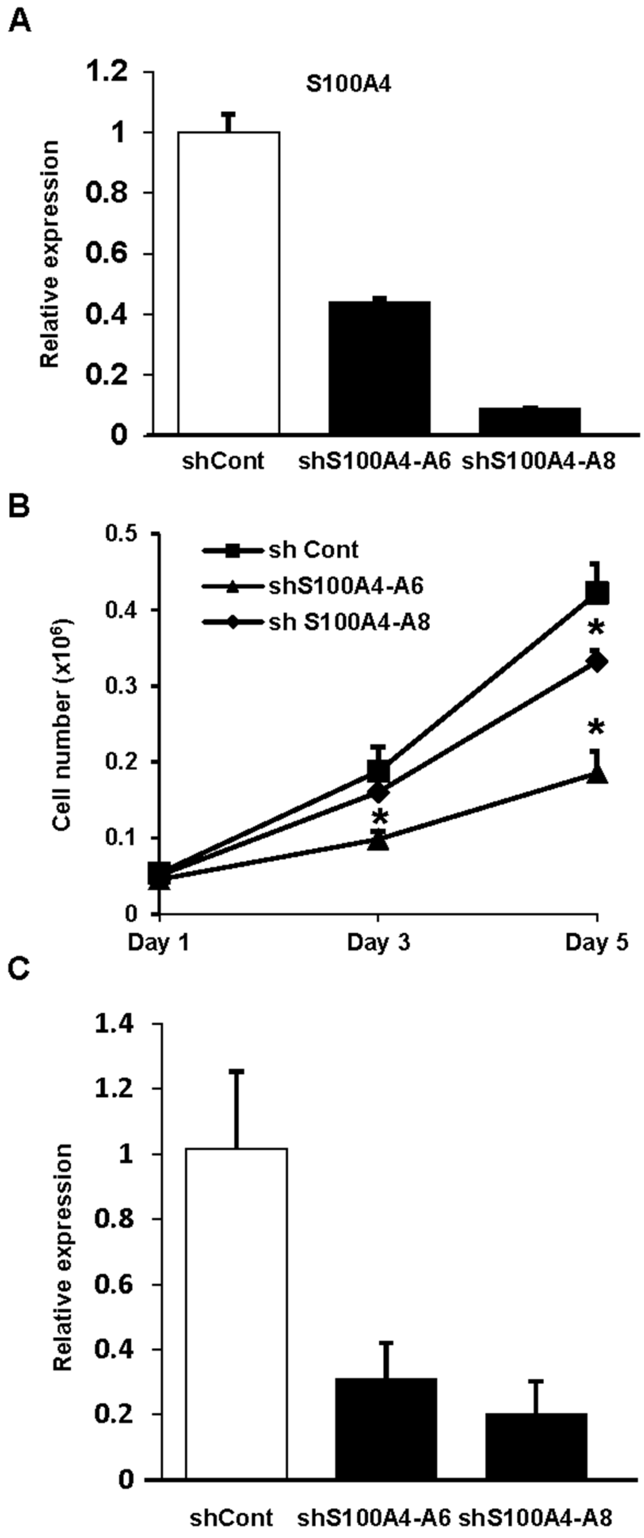

Supplementary Figure S2: Depletion S100A4 expression in H358 lung cancer cells inhibits cell proliferation and MMP9 expression. H358 cells with stable knockdown of S100A4 by two different shRNA constructs targeting S100A4 (shS100A4-A6 and shS100A4-A8) or expressing a non-targeting control (shCont) were generated. **A.** S100A4 expression was assessed by Q-PCR. **B.** Cell proliferation in standard culture was assessed by direct cell count. **C.** MMP9 expression was assessed by Q-PCR Representative data from three experiments are shown. Error bars represent the SD of the mean from three replicates. * indicates $p<0.05$.

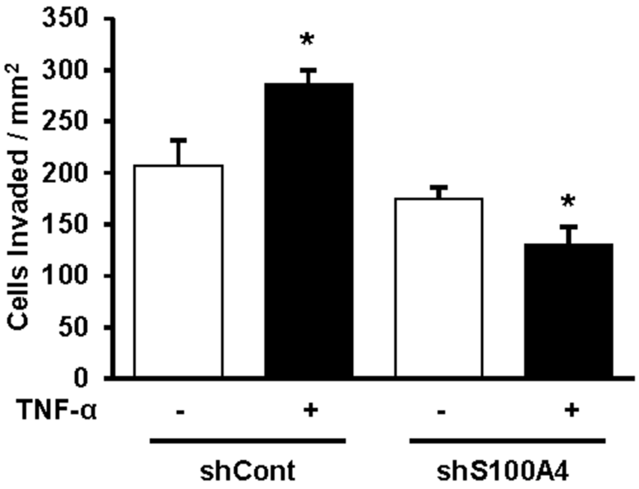

Supplementary Figure S3: Inhibition of S100A4 decreases lung cancer cells invasion toward TNF-α. A549 cells with stable knockdown of S100A4 by shRNA targeting S100A4 (shS100A4) or expressing a non-targeting control (shCont) were trypsinized, rinsed, and allowed to invade Matrigel (10 µg) toward BSA or 5 ng/ml TNF-α overnight. Representative data from three experiments are shown. Error bars represent the SD of the mean from three replicates. * indicates $p<0.05$.

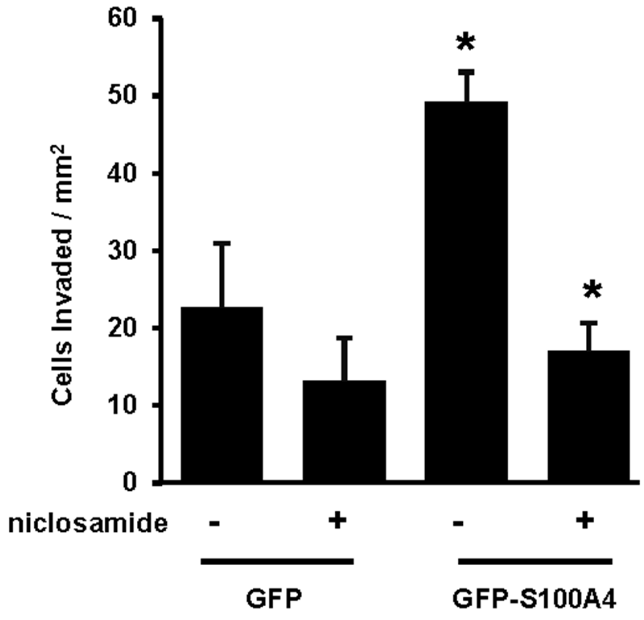

Supplementary Figure S4: Niclosamide inhibits cell invasion mediated by S100A4 overexpression in H1299 cells. H1299 cells, stably transfected with pIRES-GFP-S100A4 (GFP-S100A4) or pIRES-GFP alone (GFP) were treated with 1μM niclosamide for 48 hrs, trypsinized and assessed for Matrigel invasion overnight in the presence or absence of niclosamide. Representative data from three experiments are shown. Error bars represent the SD of the mean from three replicates. * indicates p<0.05.

Supplementary Table S1: Lung cancer cell lines tested

Cell lines	Subtype	EGFR	P53	K-ras	S100A4
A549	ADC	wt	wt	mut	+
EKVX	ADC	wt	mut	wt	+
H358	ADC	wt	null	mut	+
H838	ADC	wt	wt	wt #	+
H460	LCC	wt	wt	mut	+
Hop92	LCC	wt	mut	wt	+
H23	ADC	wt	mut	mut	-
Hop62	ADC	wt	mut	mut	-
H322 M	ADC	wt	mut	wt	-
H522	ADC	wt	mut	wt	-
H810	NSCLC§	wt	? *	wt	-
H1299	ADC	wt	null	mut	-
H157	NSCLC§	wt	? *	mut	-
H82	SCLC	wt	wt	wt	-

§ not classified into subtype
* inconsistent
# amplification

Supplementary Table S2: S100A4 expression according to histologic subtype

	S100A4 High (>2.5)	S100A4 Low (≤2.5)
**Total**	**26/212 (12%)**	**(186/212) 88%**
**Histologic Type:**		
Adenocarcinoma	17/81 (21%)	64/81 (79%)
Squamous cell carcinoma	6/100 (6%)	94/100 (94%)
**Other histologic types:**		
Poorly differentiated	2/12 (17%)	10/12 (83%)
Adenosquamous	1/12 (8%)	11/12 (92%)
Mixed histology	0/2 (0%)	2/2 (100%)
Large cell neuroendocrine	0/2 (0%)	2/2 (100%)
Pleomorphic carcinoma	0/1 (0%)	1/1 (100%)
Giant cell carcinoma	0/1 (0%)	1/1 (100%)
Sarcomatoid carcinoma	0/1 (0%)	1/1 (100%)
